# The viral proteins of influenza A virus competitively bind to TRIM31 with MAVS to fine-tune the antiviral innate immunity

**DOI:** 10.1128/jvi.01893-25

**Published:** 2025-11-26

**Authors:** Jiaxin Huang, Shuai Xu, Junwen Liu, Qian Wang, Lu Han, Mengyao Ji, Caoqi Lei, Qiyun Zhu, Hualan Chen

**Affiliations:** 1 College of Veterinary Medicine, Gansu Agricultural University 739715, Lanzhou, People's Republic of China; 2 State Key Laboratory for Animal Disease Control and Prevention, Lanzhou Veterinary Research Institute, Chinese Academy of Agricultural Sciences 111658 , Lanzhou, People's Republic of China; 3 School of Basic Medical Sciences, Lanzhou University 426140, Lanzhou, People's Republic of China; 4 State Key Laboratory for Animal Disease Control and Prevention, Harbin Veterinary Research Institute, Chinese Academy of Agricultural Sciences 687216 , Harbin, People's Republic of China; University of Minnesota Twin Cities, Minneapolis, Minnesota, USA

**Keywords:** influenza A virus, TRIM31, ubiquitination, viral protein, antiviral innate immunity

## Abstract

The influenza A virus (IAV) continues to pose a serious threat to animals and humans, making it urgent to reveal more about IAV-host interactions. Tripartite motif protein 31 (TRIM31), an E3 ubiquitin ligase, has been identified as an agonist of the type-I interferon (IFN-I) response against RNA viruses by targeting mitochondrial antiviral signaling protein (MAVS). Here, we demonstrated that TRIM31 plays critical and novel roles in the life cycle of IAV. TRIM31 promoted the IFN-I signaling induced by IAV; however, it was surprisingly found that TRIM31 does not affect IAV replication. Instead, IAV replication was significantly promoted by TRIM31 in MAVS- or interferon receptor-deficient cells, suggesting TRIM31 may facilitate IAV replication in an interferon-independent manner. Mechanistically, TRIM31 interacted specifically with the basic polymerase 1 (PB1), acidic polymerase (PA), and hemagglutinin (HA) proteins of different subtypes of IAV. The interaction between TRIM31 and the PB1, PA, and HA proteins enhances the stability and polymerase and membrane fusion activities of these viral proteins by catalyzing the K63-linked ubiquitination. Further, the PB1, PA, and HA proteins competitively bind to TRIM31 for IAV replication, leading to the attenuation of the TRIM31-MVAS complex-mediated IFN-I signaling activation. Therefore, the antiviral and proviral effects of TRIM31 reach a balance in IAV-infected cells, resulting in no significant impact on IAV replication. Our novel findings revealed an IAV-specific mechanism that IAV exploits TRIM31 to fine-tune the antiviral innate response and maintain the homeostasis of viral replication.

**IMPORTANCE:**

During the long-term symbiosis with the host, IAVs have evolved a series of unique mechanisms to adapt to the host and support their own replication. The MAVS-mediated IFN-I signaling pathway is crucial for host cells to defend against RNA virus invasion, with TRIM31 functioning as a specific agonist for the activation of IFN-I antiviral response. In the present study, we demonstrated that IAV exploits TRIM31 to promote the stability and activity of viral proteins and reduces the positive effect of TRIM31 on the IFN-I response, thereby preventing TRIM31 from inhibiting IAV replication. Therefore, our results revealed a novel mechanism employed by IAV to adapt to host antiviral response and expanded our understanding of virus–host interactions.

## INTRODUCTION

Influenza A virus (IAV) is an enveloped, negative-sense, single-stranded RNA virus that causes respiratory diseases ranging from mild to severe. IAV poses a significant threat to both human and animal health, causing annual seasonal epidemics and sporadic pandemic outbreaks (1–3). According to reports from the World Health Organization (WHO), the influenza epidemics result in 3–5 million cases of severe illness and approximately 290,000-650,000 deaths worldwide each year (4). During the virus life cycle, IAV employs various strategies to facilitate viral replication and spread, including counteracting host immune responses (5, 6), hijacking energy and substance metabolism (7, 8), and modulating cell death (9, 10). The virus–host interactions serve as a critical foundation for the propagation, transmission, and adaptation of viruses. Consequently, elucidating the molecular mechanisms underlying IAV–host interactions is essential for developing both prophylactic and therapeutic strategies against IAV.

Ubiquitination is a vital post-translational modification (PTM) involved in various mechanisms that maintain intracellular homeostasis. The cellular ubiquitin ligases (E3s), in cooperation with ubiquitin-activating enzymes (E1s) and ubiquitin-conjugating enzymes (E2s), are responsible for the ubiquitination of substrate proteins (11). Ubiquitin chains are organized by the conjugation of different ubiquitin residues through a variety of lineages (12). The well-described proteasomal degradation machinery is activated by K48-linked polyubiquitination (13). K63-linked polyubiquitination has been demonstrated to play a crucial role in activating and stabilizing proteins involved in vital cellular processes (14–16). Numerous studies have demonstrated the pivotal function of ubiquitination in virus–host interactions. For example, E3 ligases, such as tripartite motif 25 (TRIM25), TRIM9, and ring finger protein 5 (RNF5), have been reported to regulate the antiviral response by increasing the ubiquitination of key proteins in the interferon pathway (17–19). Further, several E3 ligases, including TRIM14, TRIM21, and Bcl2-associated athanogene 6 (BAG6), have been shown to directly target viral proteins, thereby regulating their stability and/or their interactions with other viral proteins (20–22).

The TRIM protein family is one of the largest subfamilies of E3 ubiquitin ligases, comprising over 80 members (23). As a member of the TRIM-containing proteins, tripartite motif 31 (TRIM31) has been implicated in a variety of pathological processes, including inflammatory diseases, protein quality control, autophagy, viral infection, and carcinoma development (24–27). Upon RNA virus infection, TRIM31 promotes the K63-linked polyubiquitination of mitochondrial antiviral signaling protein (MAVS), thereby facilitating the formation of MAVS prion-like aggregates and MAVS-mediated type I IFN (IFN-I) signaling, and inhibiting the replication of RNA viruses, such as Sendai virus (SeV) and vesicular stomatitis virus (VSV) (26). Therefore, TRIM31 is a positive regulator of the MAVS-mediated IFN-I response.

In the present study, we revealed that TRIM31 potentiates the IFN-I response induced by IAV. Whereas, TRIM31 is exploited by IAV to enhance the stability of viral PB1, PA, and HA proteins by catalyzing K63-linked ubiquitination. The stabilized PB1, PA, and HA proteins of IAV competitively bind to TRIM31 with MAVS, thereby alleviating the innate immune response. Our data demonstrated that IAV exploits TRIM31 to fine-tune its positive effect on the IFN-I response for the homeostasis of viral replication.

## RESULTS

### TRIM31 potentiates the IFN-I response induced by IAV

**Fig 1:**
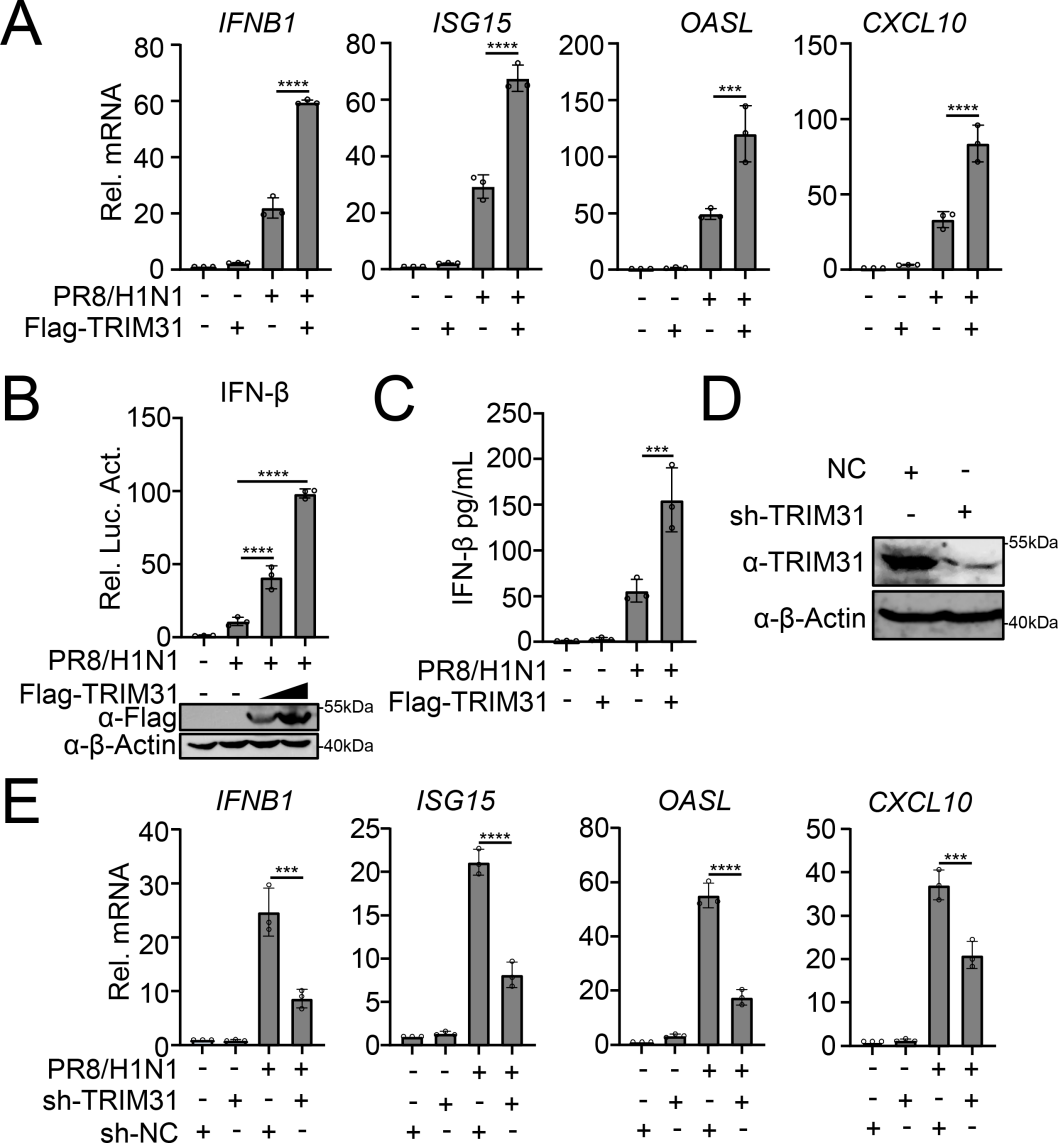
TRIM31 potentiates the IFN-I response induced by IAV. (**A**) A549 cells were transfected with Flag-TRIM31 and empty vector (EV) for 24 h. The cells were then infected with PR8/H1N1 at a multiplicity of infection (MOI) of 3 before qPCR assays were performed. (**B**) HEK293T cells were transfected with IFN-β-Luc, TK, Flag-TRIM31, or EV for 24 h and then infected with PR8/H1N1 for 12 h. The supernatants were detected in luciferase assays. (**C**) A549 cells were transfected with Flag-TRIM31 or EV for 24 h and induced by PR8/H1N1 infection. The concentration of IFN-β in the supernatants was detected by enzyme-linked immunosorbent assay (ELISA). (**D**) Sh-TRIM31 or sh-NC A549 cells were harvested, and Western blotting analyses were performed with the indicated antibodies. (**E**) Sh-NC or sh-TRIM31 A549 cells were infected with PR8/H1N1 at an MOI of 3 for 24 h before qPCR assays were performed. The data represent three independent experiments; bars represent the mean ± SDs of the three independent experiments (*n* = 3). *** *P* <0.001, **** *P* <0.0001.

Based on the promotion of TRIM31 to the aggregates of MAVS and SeV- or VSV-induced IFN-I responses (26), we hypothesized that TRIM31 may also promote the IAV-induced IFN-I response. As shown in Fig. 1A, overexpression of TRIM31 increased the transcription of the interferon beta 1 (*IFNB1*), IFN-stimulated gene 15 (*ISG15*), oligoadenylate (*OASL*), and C-X-C motif chemokine 10 (*CXCL10*) genes in IAV-infected cells. Further, overexpressed TRIM31 enhanced IAV-induced activation of the IFN-β promoter in a dose-dependent manner (Fig. 1B). Similarly, TRIM31 overexpression led to an augmentation in the secretion of IFN-β following IAV, exhibiting a dose-dependent relationship (Fig. 1C). Conversely, silencing TRIM31 resulted in the downregulation of the transcription of the *IFNB1*, *ISG15*, *OASL*, and *CXCL10* genes induced by IAV (Fig. 1D and E). These data suggest that TRIM31 upregulates the IAV-triggered IFN-I signaling pathway.

**Fig 2:**
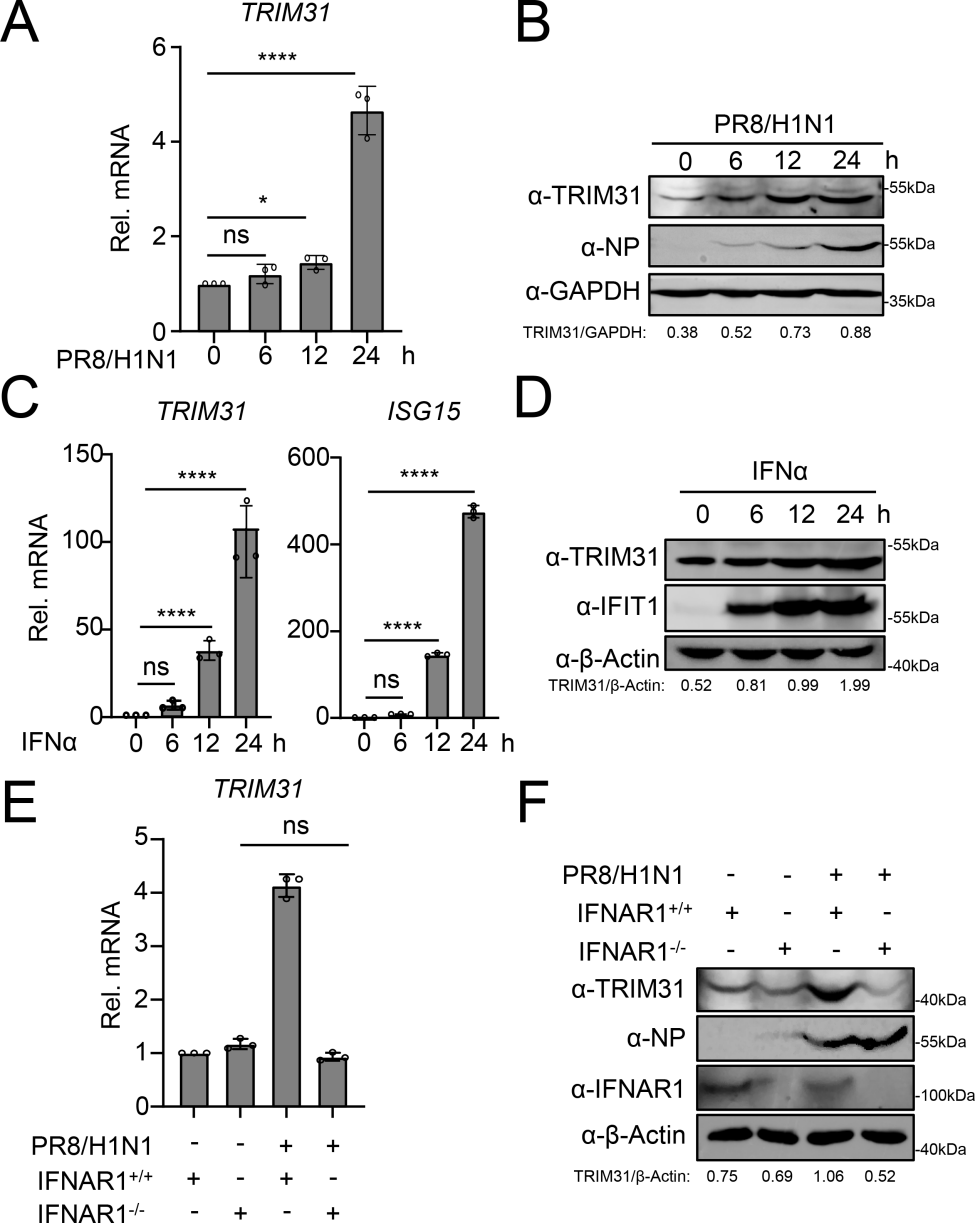
TRIM31 is an interferon-stimulated gene. (**A**) A549 cells were infected with PR8/H1N1 at an MOI of 3 for the indicated times before qPCR assays. (**B**) HEK293T cells were infected with PR8/H1N1 at an MOI of 3 for the indicated times before Western blotting analysis. The intensities of the TRIM31 protein bands were determined and normalized to GAPDH using ImageJ. (**C–D**) A549 cells were treated with IFNα (50 ng/mL) for the indicated times before qPCR and Western blotting analysis. The intensities of the TRIM31 protein bands were determined and normalized to β-actin using ImageJ. (**E–F**) The IFNAR1-deficient (IFNAR1^-/-^) or control (IFNAR1^+/+^) A549 cells were infected with PR8/H1N1 at an MOI of 3 for 12 h before qPCR and Western blotting analysis. The intensities of the TRIM31 protein bands were determined and normalized to β-actin using ImageJ. The data represent three independent experiments; bars represent the mean ± SDs of the three independent experiments (*n* = 3). * *P* < 0.05, **** *P* <0.0001, ns, no significant difference.

We further detected the expression of TRIM31 following IAV infection. As shown in Fig. 2A and B, the mRNA and protein of TRIM31 were both upregulated upon IAV infection, which suggested that the transcription of the TRIM31 gene was induced by IAV infection. The online tools, such as hTFtarget (28) and JASPAR (29), were used to screen the transcription factors that bind to the TRIM31 promoter. The signal transducer and activator of transcription 1 (STAT1), as a well-known transcription factor within the IFN signaling pathway, was found to recognize several motifs within the TRIM31 promoter (data not shown), suggesting that TRIM31 may be an interferon-stimulated gene (ISG). To verify this, the mRNA expression of TRIM31 in cells was elevated following IFNα treatment, as well as the expression of TRIM31 protein (Fig. 2C and D). In addition, the IAV infection failed to enhance the expression of TRIM31 in interferon alpha and beta receptor subunit 1 (IFNAR1)-deficient cells (Fig. 2E and F). These data indicate that TRIM31 is a novel ISG that promotes the innate immune response.

### TRIM31 enhances IAV replication in VERO and MAVS-deficient cells

**Fig 3:**
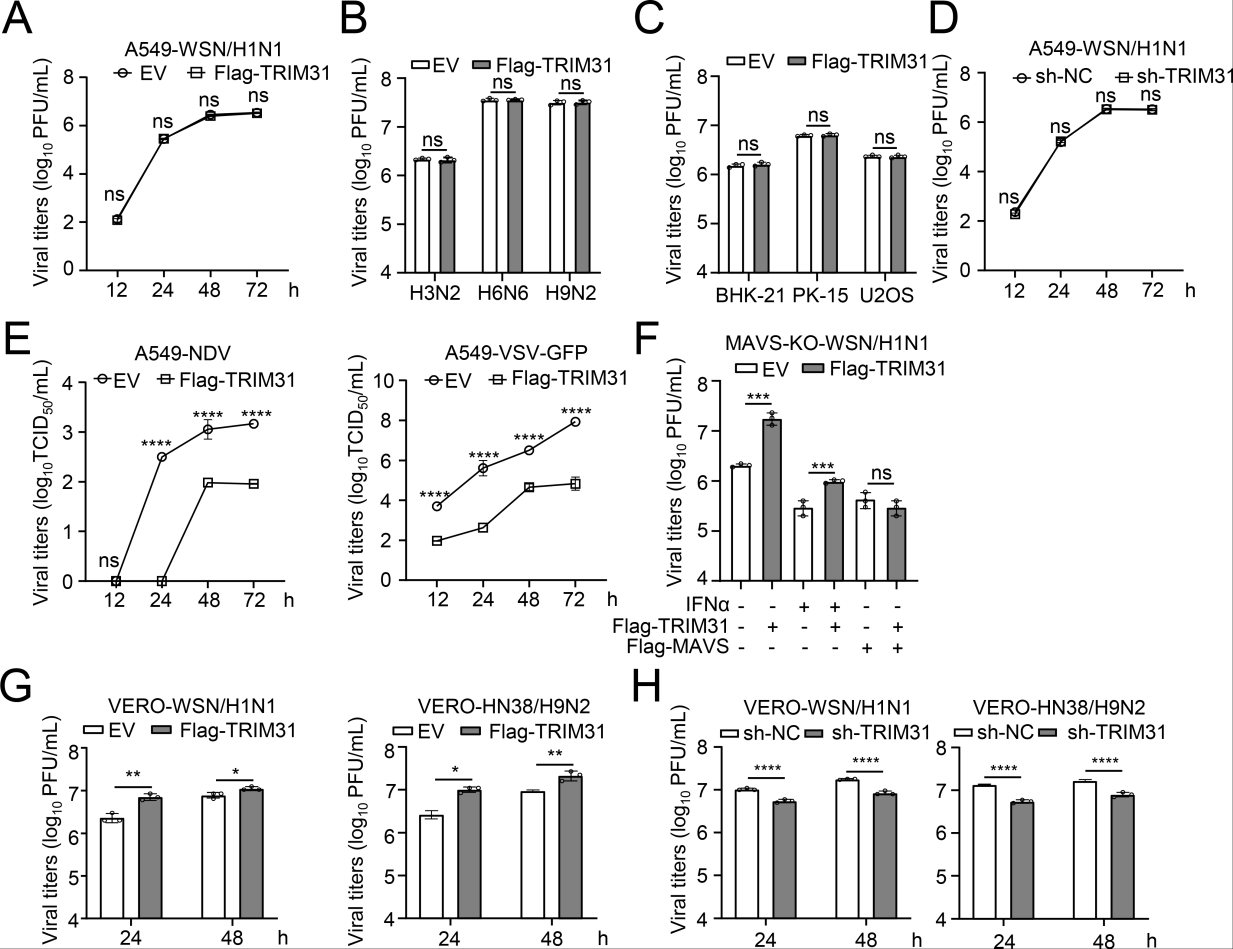
TRIM31 enhances the replication of IAV in IFN-deficient VERO cells. (**A**) A549 cells were transfected with either the Flag-TRIM31 expression plasmid or EV for 24 h before being infected with WSN/H1N1 at an MOI of 0.01. At the indicated times post-infection, the supernatants containing viral particles were assessed using a plaque-forming unit (PFU) assay. (**B**) A549 cells were transfected with either the Flag-TRIM31 expression plasmid or EV. At 24 h post-transfection, the cells were infected with TA5/H3N2, HN4/H6N6, or HN38/H9N2 viruses at an MOI of 0.01 for 48 h. Supernatants were collected and assessed using a PFU assay. (**C**) BHK-21, PK-15, and U2OS cells were transfected with either Flag-TRIM31 or EV for 24 h before the cells were infected with WSN/H1N1 virus at an MOI of 0.01. At 48 h post-infection, the supernatants containing viral particles were assessed using a PFU assay. (**D**) Sh-TRIM31 or sh-NC A549 cells were infected with WSN/H1N1 at an MOI of 0.01 for 48 h, and the supernatants containing viral particles were collected and assessed using a PFU assay. (**E**) A549 cells were transfected with either Flag-TRIM31 or EV for 24 h before being infected with NDV or VSV-GFP virus at an MOI of 0.01. At the indicated times post-infection, the supernatants containing viral particles were assessed using a TCID_50_ assay. (**F**) MAVS-knockout A549 cells were transfected with Flag-TRIM31 and/or Flag-MAVS for 24 h and then infected with WSN/H1N1 at an MOI of 0.01, along with IFNα (50 ng/mL) treatment or not. At 24 h post-infection, the supernatants containing viral particles were assessed using a PFU assay. (**G**) VERO cells were transfected with either Flag-TRIM31 or EV. At 24 h post-transfection, the cells were infected with WSN/H1N1 and HN38/H9N2 viruses at an MOI of 0.01. At the indicated times post-infection, the supernatants containing viral particles were assessed using a PFU assay. (**H**) Sh-TRIM31 or sh-NC VERO cells were infected with WSN/H1N1 and HN38/H9N2 viruses at an MOI of 0.01 for 48 h, and the supernatants containing viral particles were assessed using a PFU assay. The data represent three independent experiments; bars represent the means ± SDs of the three independent experiments (*n* = 3). * *P* < 0.05,** *P* <0.01,*** *P* <0.001, **** *P* <0.0001, ns, no significant difference.

As TRIM31 upregulates the IFN-I response, it can be inferred that TRIM31 restricts IAV replication. Interestingly, TRIM31 overexpression did not impact the replication of H1N1 IAV in A549 cells (Fig. 3A), nor that of H3N2, H6N6, or H9N2 IAVs (Fig. 3B). In cells derived from different species (hamster BHK-21 cells, swine PK-15 cells, and human U2OS cells), IAV replication remained unaffected by TRIM31 overexpression (Fig. 3C). In addition, knockdown of TRIM31 had no effect on IAV replication (Fig. 3D). However, overexpressed TRIM31 significantly inhibited the replication of other RNA viruses, such as the Newcastle disease virus (NDV) and VSV (Fig. 3E), which is consistent with a previous study (26). As TRIM31 potentiates IFN induction by mediating the polyubiquitination and oligomerization of MAVS, we generated MAVS-deficient cells to investigate the impact of TRIM31 on IAV replication. In MAVS-deficient cells, TRIM31 overexpression promoted IAV replication, even with IFNα treatment restricting virus replication (Fig. 3F). In VERO cells (an interferon receptor-deficient cell line), TRIM31 overexpression promoted the replication of H1N1 and H9N2 viruses, whereas TRIM31 knockdown inhibited virus replication (Fig. 3G and H). These results indicate that TRIM31 promotes IAV replication in the absence of MAVS or the interferon pathway, suggesting that TRIM31 may facilitate IAV replication in an IFN-independent manner.

### TRIM31 interacts with and stabilizes PB1, PA, and HA of IAV

**Fig 4:**
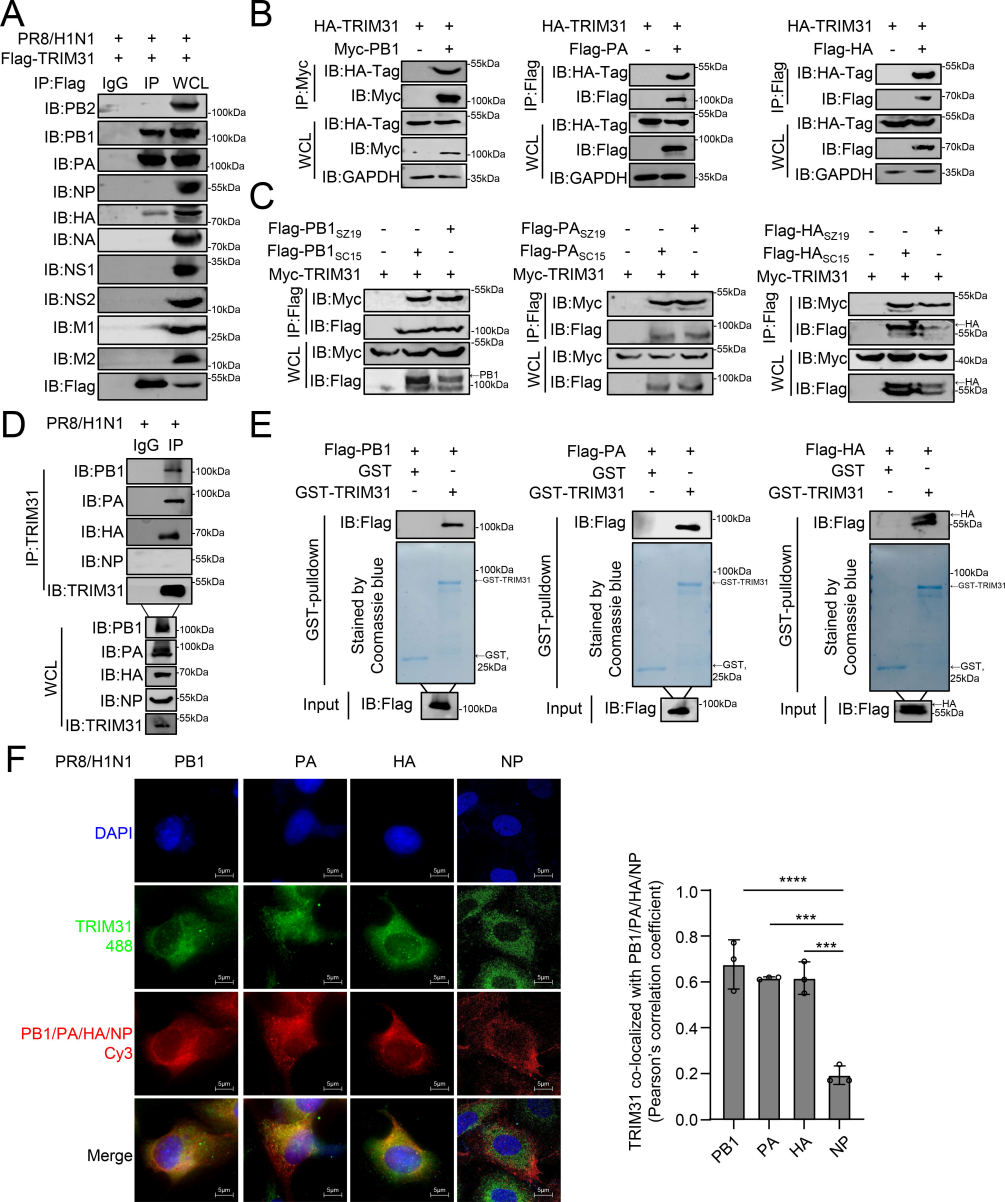
TRIM31 interacts with the PB1, PA, and HA proteins of IAV. (**A**) HEK293T cells were transfected with Flag-TRIM31 for 24 h and then infected with PR8/H1N1 virus at an MOI of 0.1 for 24 h before co-IP and immunoblot analysis. (**B**) HEK293T cells were transfected with the indicated plasmids for 24 h before co-IP and immunoblot analysis. (**C**) HEK293T cells were transfected with the indicated plasmids for 24 h before co-IP and immunoblot analysis. (**D**) HEK293T cells were infected with PR8/H1N1 for 24 h before co-IP and Western blotting analysis. (**E**) Purified GST-TRIM31 was used to pull down Flag-tagged PB1, PA, and HA_PR8_. (**F**) U2OS cells were transfected with Flag-TRIM31 for 24 h and subsequently infected with PR8/H1N1 for 24 h. The right panel shows the quantification of Pearson’s colocalization coefficient between viral proteins and TRIM31. The data represent three independent experiments; bars represent the means ± SDs of the three independent experiments (*n* = 3). *** *P* <0.001, **** *P* <0.0001.

Given that TRIM31 promotes the IFN-I response induced by IAV but facilitates the replication of IAV in cells lacking interferon receptors, we hypothesized that TRIM31 might facilitate IAV replication through an alternative mechanism. As TRIM31 is an E3 ligase, we speculated that it may regulate the ubiquitination of viral proteins. An endogenous co-immunoprecipitation (co-IP) assay revealed that TRIM31 is associated with the PB1, PA, and HA proteins of the H1N1 virus but not with other viral proteins, such as PB2, NA, and M1 (Fig. 4A). In a confirmatory experiment, TRIM31 interacted with Flag-tagged PB1, PA, and HA (Fig. 4B). Furthermore, TRIM31 interacted with overexpressed PB1, PA, and HA proteins derived from H5N6 and H7N9 IAVs (Fig. 4C). Consistently, the PB1, PA, and HA proteins were found to specifically interact with endogenous TRIM31 in IAV-infected cells (Fig. 4D). In addition, a glutathione S-transferase (GST) pulldown assay demonstrated that TRIM31 directly interacts with PB1, PA, and HA *in vitro* (Fig. 4E). Moreover, TRIM31 co-localized with PB1, PA, and HA, but not NP, in the cytoplasm of virus-infected cells (Fig. 4F). These data demonstrate that TRIM31 specifically and directly associates with the PB1, PA, and HA proteins of IAV.

**Fig 5:**
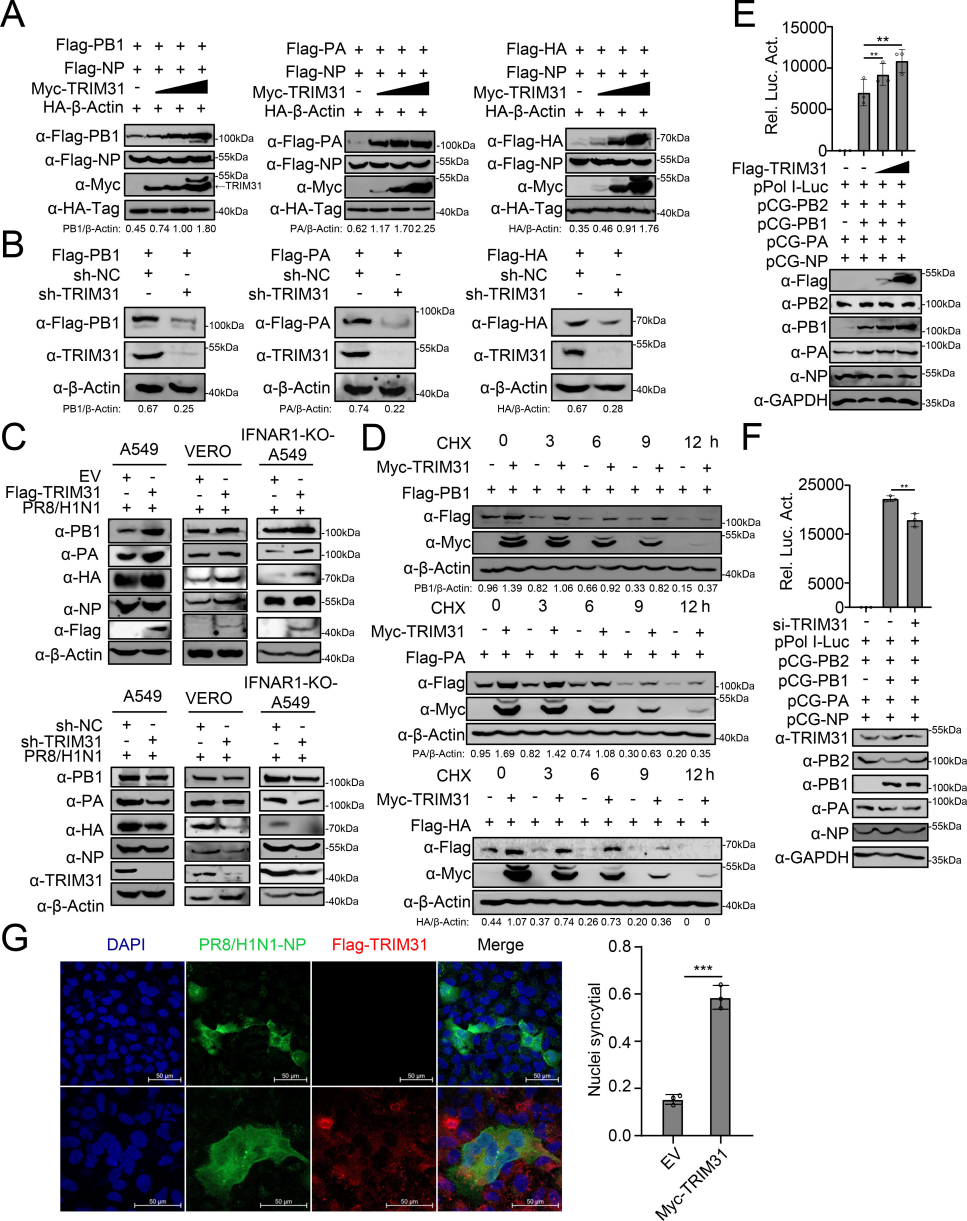
TRIM31 stabilizes the PB1, PA, and HA proteins of IAV. (**A**) HEK293T cells were transfected with HA-β-Actin, along with Myc-NP, increasing amounts of Myc-TRIM31, and Flag-PB1, Flag-PA, or Flag-HA for 24 h before Western blotting analysis. The intensities of the viral protein bands were determined and normalized to β-actin using ImageJ. (**B**) Sh-TRIM31 and sh-NC HEK293T cells were transfected with Flag-PB1, Flag-PA, or Flag-HA for 24 h before Western blotting analysis. The intensities of the viral protein bands were determined and normalized to β-actin using ImageJ. (**C**) A549, VERO, or IFNAR1^-/-^ cells were transfected with Flag-TRIM31 or EV for 24 h and then infected with WSN/H1N1 for 24 h before Western blotting analysis. (**D**) HEK293T cells were transfected with the indicated plasmids for 24 h and then treated with CHX (100 µg/ mL) before harvest. Total proteins were extracted and detected by Western blotting analysis. The intensities of the viral protein bands were determined and normalized to β-actin using ImageJ. (**E–F**) HEK293T cells were transfected with the polymerase proteins pCG-PB2, PB1, PA, NP, pPol I-Luc, and Flag-TRIM31 or si-TRIM31. At 24 h post-transfection, the cells were collected for luciferase assays and Western blotting analysis. (**G**) A549 cells were transfected with Flag-TRIM31 or EV for 24 h and then infected with PR8/H1N1 at an MOI of 3 for 12 h. Cells were then treated with TPCK-treated trypsin for 15 min, followed by citric acid (pH 5.0) for 1 h. Syncytium formation was observed by immunofluorescence with the indicated antibodies. The right panel shows the quantitative analysis of syncytia. The data represent three independent experiments; bars represent the means ± SDs of the three independent experiments (*n* = 3). ** *P* <0.01,*** *P* <0.001.

Studies have shown that TRIM31-mediated ubiquitination regulates the stability of substrate proteins (26, 30, 31). As shown in Fig. 5A, TRIM31 increased the expression of PB1, PA, and HA in a dose-dependent manner, while exerting no effect on NP expression. Knockdown of TRIM31 downregulated the expression of PB1, PA, and HA (Fig. 5B). Overexpression of TRIM31 upregulated the expression of PB1, PA, and HA in IAV-infected A549 cells, as well as in VERO and IFNAR1-knockout A549 cells (Fig. 5C). Using cycloheximide (CHX), an inhibitor of eukaryotic translation elongation, to block protein translation, it was confirmed that TRIM31 prolonged the half-lives of PB1, PA, and HA (Fig. 5D). PB1 and PA are vital subunits of the viral polymerase complex and contribute to the transcription and replication of the viral genome. A minigenome assay showed that TRIM31 overexpression enhanced the viral polymerase activity in a dose-dependent manner, whereas knockdown of TRIM31 reduced the viral polymerase activity (Fig. 5E and F). One of the major functions of HA in the IAV life cycle is to mediate the fusion between the viral and cellular membranes. The membrane fusion assay demonstrated that overexpression of TRIM31 enhanced the HA-mediated cell-to-cell membrane fusion (Fig. 5G). These results indicate that TRIM31 strengthens the stability and activity of PB1, PA, and HA proteins in cells.

### TRIM31 catalyzes the K63-linked ubiquitination of PB1, PA, and HA

**Fig 6:**
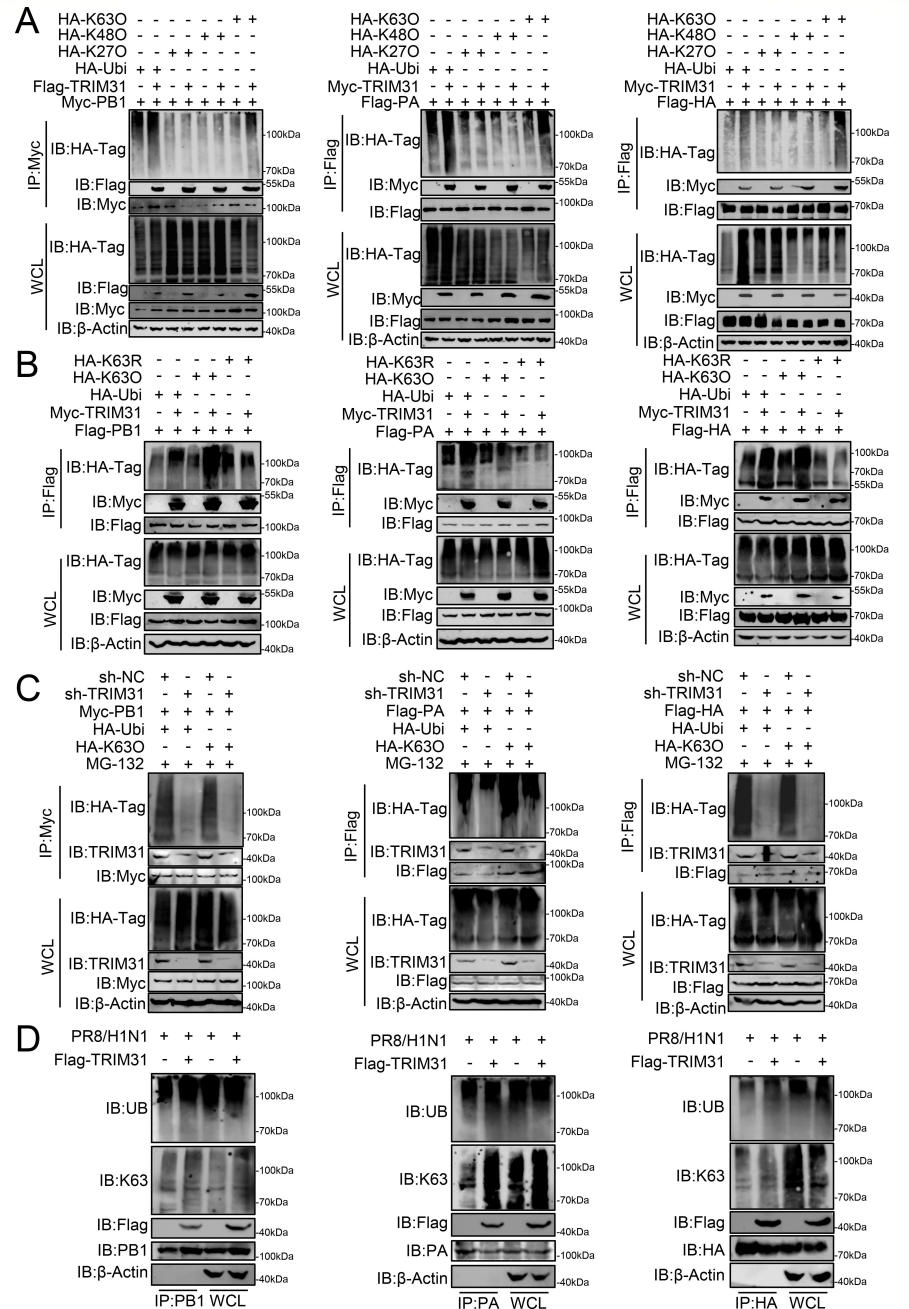
TRIM31 promotes the K63-linked ubiquitination of PB1, PA, and HA. (**A**) HEK293T cells were transfected with plasmids expressing Myc-TRIM31, Flag-PB1, Flag-PA, or Flag-HA, HA-ubiquitin, or its mutants (K-O, in which all lysine residues but one were simultaneously mutated to arginine [K-only]) for 24 h and then subjected to co-IP and western blotting analysis with the indicated antibodies. (**B**) HEK293T cells were transfected with Flag-PB1, Flag-PA, or Flag-HA_PR8_, HA-K63O, and HA-K63R (in which the K63 lysine residues were mutated to arginine) together with Myc-TRIM31 plasmids for 24 h. The cells were then used for ubiquitination assays. (**C**) Sh-TRIM31 and sh-NC HEK293T cells were transfected with HA-K63O and Flag-PB1, Flag-PA, or Flag-HA_PR8_ plasmids for 24 h, and then subjected to co-IP and Western blotting analysis with the indicated antibodies. (**D**) HEK293T cells were transfected with Flag-TRIM31 or EV for 24 h, and then infected with WSN/H1N1 for 24 h. The cells were then used for ubiquitination assays. The data represent three independent experiments; bars represent the means ± SDs of the three independent experiments (*n* = 3).

Next, we seek to demonstrate how TRIM31 contributes to the stabilization of PB1, PA, and HA. In a ubiquitination assay, TRIM31 overexpression was found to significantly increase the K63-linked ubiquitination of PB1, PA, and HA but not K27- or K48-linked ubiquitination (Fig. 6A). Furthermore, TRIM31 overexpression facilitated the ubiquitination of K63O, which contains only one lysine at position 63 of ubiquitin, to PB1, PA, and HA, but not K63R (in which the K63 lysine residue is mutated to arginine) (Fig. 6B). Knockdown of TRIM31 led to a decrease in the K63-linked ubiquitination of PB1, PA, and HA (Fig. 6C). Similarly, TRIM31 overexpression led to an enhancement of K63-linked ubiquitination of PB1, PA, and HA in IAV-infected cells (Fig. 6D). In addition, TRIM31 promoted the K63-linked ubiquitination of PB1, PA, and HA derived from H5N6 and H7N9 IAVs (Fig. S1). Taken together, these data indicate that TRIM31 increases the K63-linked ubiquitination of PB1, PA, and HA.

**Fig 7:**
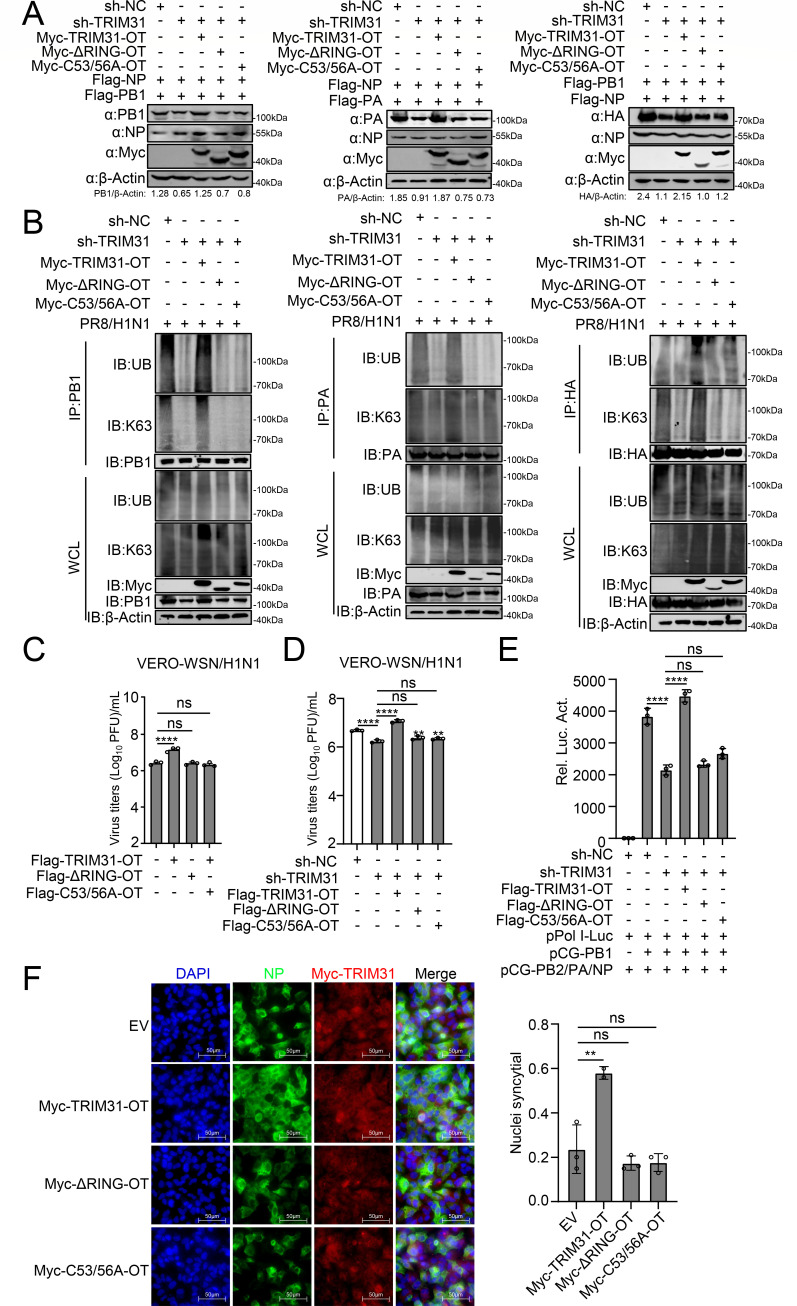
The ubiquitin ligase activity is critical for TRIM31 to stabilize the viral proteins of IAV. (**A**) Sh-TRIM31 or sh-NC HEK293T cells were transfected with the indicated plasmids for 24 h before Western blotting analysis. The intensities of the viral protein bands were determined and normalized to β-actin using ImageJ. (**B**) Sh-TRIM31 or sh-NC HEK293T cells were transfected with TRIM31-OT, TRIM31-ΔRING-OT, or TRIM31-C53/56A-OT for 24 h before being infected with PR8/H1N1. At 24 h post infection, the cells were then used for ubiquitination assays. (**C**) VERO cells were transfected with TRIM31-OT, TRIM31-ΔRING-OT, or TRIM31-C53/56A-OT for 24 h before infected with WSN/H1N1 virus at an MOI of 0.01 for 48 h, and the supernatants containing viral particles were assessed using a PFU assay. (**D**) Sh-TRIM31 or sh-NC VERO cells were transfected with TRIM31-OT, TRIM31-ΔRING-OT, or TRIM31-C53/56A-OT for 24 h before infected with WSN/H1N1 virus at an MOI of 0.01 for 48 h, and the supernatants containing viral particles were assessed using a PFU assay. (**E**) Sh-TRIM31 or sh-NC HEK293T cells were transfected with the polymerase proteins pCG-PB2, PB1, PA, NP, and pPol I-Luc, along with TRIM31-OT, TRIM31-ΔRING-OT, or TRIM31-C53/56A-OT. At 24 h post-transfection, the cells were collected for luciferase assays. (**F**) A549 cells were transfected with TRIM31-OT, TRIM31-ΔRING-OT, or TRIM31-C53/56A-OT, or EV for 24 h and then infected with PR8/H1N1 at an MOI of 3 for 12 h. Cells were then treated with TPCK-treated trypsin for 15 min, followed by citric acid (pH 5.0) for 1 h. Syncytium formation was observed by immunofluorescence with indicated antibodies. The right panel showed the quantitative analysis of syncytia. The data represent three independent experiments; bars represent the means ± SDs of the three independent experiments (*n* = 3). ** *P* <0.01, **** *P* <0.0001, ns, no significant difference.

Prior studies have demonstrated that the RING domain of TRIM31 is indispensable for E3 ligase activity, with the critical cysteine residues at positions 53 and 56 (C53/56) located within this domain. TRIM31-ΔRING, which lacks the RING domain, and the C53/56A mutation, in which the critical cysteine residues were replaced with alanine, both lose the ubiquitin ligase activity of TRIM31 (26, 30). To investigate whether TRIM31-mediated stabilization of viral proteins and promotion of viral replication depends on E3 ligase activity, we constructed plasmids expressing TRIM31-ΔRING and TRIM31-C53/56A. To resist the interference effect of sh-TRIM31, the sh-TRIM31 off-target nonsense mutants of wild-type TRIM31, TRIM31-ΔRING, and TRIM31-C53/56A were constructed, i.e., TRIM31-OT, TRIM31-ΔRING-OT, and TRIM31-C53/56A-OT (Fig. S2A). As shown in Fig. S2B, the truncation of the RING domain and the C53/56A mutation did not affect the interaction of TRIM31 with PB1, PA, and HA. However, wild-type TRIM31 restored the decrease of PB1, PA, and HA proteins caused by TRIM31 knockdown, whereas TRIM31-ΔRING and TRIM31-C53/56A did not (Fig. 7A). Knockdown of TRIM31 led to a decrease in the K63-linked ubiquitination of PB1, PA, and HA in both HEK293T and MAVS-deficient HEK293T cells. This decrease was restored by wild-type TRIM31 but not by TRIM31-ΔRING or TRIM31-C53/56A (Fig. 7B; Fig. S2C). In VERO cells, overexpression of wild-type TRIM31 led to the promotion of IAV replication, while knockdown of TRIM31 led to the inhibition of IAV replication. TRIM31-ΔRING and TRIM31-C53/56A had no effect on IAV replication (Fig. 7C and D). Consistently, TRIM31-ΔRING and TRIM31-C53/56A failed to restore the inhibition of viral polymerase activity and HA-mediated membrane fusion caused by TRIM31 knockdown (Fig. 7E and F). These results suggest that TRIM31 catalyzes the K63-linked ubiquitination of the PB1, PA, and HA proteins via E3 ligase activity and promotes the expression and function of the viral proteins.

### Influenza viral proteins attenuate the interaction between TRIM31 and MAVS

**Fig 8:**
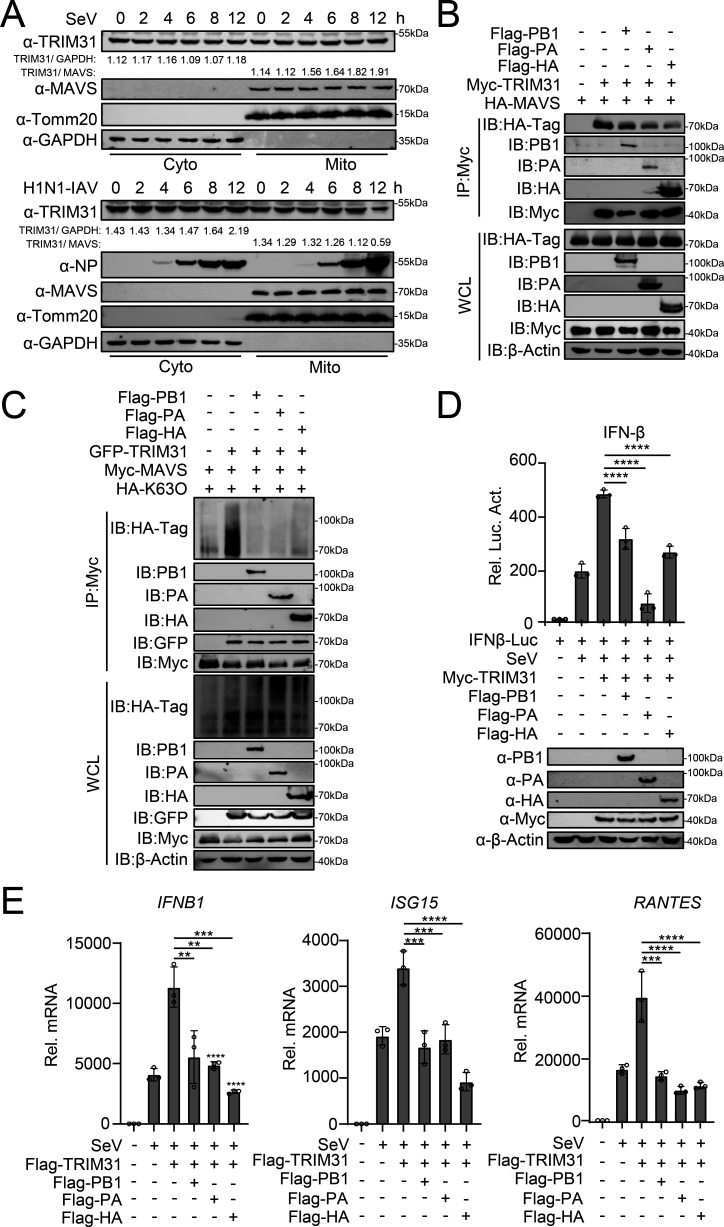
IAV proteins attenuate the interaction between TRIM31 and MAVS. (**A**) HEK293T cells were infected with PR8/H1N1 or SeV at an MOI of 3 for the indicated times. Cytoplasmic and mitochondrial fractions were purified for western blotting (Fractions: Cyto, purified cytosolic; Mito, purified mitochondria). The intensities of the TRIM31 protein bands were determined and normalized to GAPDH or MAVS using ImageJ. (**B**) HEK293T cells were transfected with HA-MAVS, Myc-TRIM31, along with Flag-PB1, Flag-PA, or Flag-HA for 24 h and then subjected to co-IP and western blotting analysis with the indicated antibodies. (**C**) HEK293T cells were transfected with the indicated plasmids for 24 h and then subjected to co-IP and Western blotting analysis. (**D**) HEK293T cells were transfected with indicated plasmids for 24 h and then infected with SeV for 12 h before luciferase assay and Western blotting analysis. (**E**) A549 cells were transfected with the indicated plasmids for 24 h and then infected with SeV for 12 h before qPCR assays were performed. The data represent three independent experiments; bars represent the means ± SDs of the three independent experiments (*n* = 3). ** *P* <0.01,*** *P* <0.001, **** *P* <0.0001.

As TRIM31 binds to and enhances the ubiquitination of both MAVS and viral proteins, so we began to illustrate the impact of viral proteins on the TRIM31–MAVS interaction and MAVS-mediated interferon responses. As MAVS is a mitochondria-located protein, TRIM31 can be recruited to mitochondria by MAVS. SeV infection has been demonstrated to increase the co-localization of TRIM31 with mitochondria (26). Consistently, the fraction of TRIM31 on mitochondria was increased following SeV infection. However, in IAV-infected cells, the fraction of TRIM31 on mitochondria was not increased; rather, it was decreased following infection with IAV (Fig. 8A). These data suggest that less TRIM31 is recruited by MAVS in IAV-infected cells than in SeV-infected cells. As a verification, overexpression of PB1, PA, and HA reduced the interaction between TRIM31 and MAVS and the TRIM31-mediated, K63-linked ubiquitination of MAVS (Fig. 8B and C). A luciferase assay revealed that PB1, PA, and HA reduced the TRIM31-mediated upregulation of the IFN-β promoter, as well as the transcription of the *IFNB1*, *ISG15*, and regulated upon activation normal T cell expressed and secreted factor (*RANTES*) genes (Fig. 8D and E). The above data revealed that IAV proteins competitively interact with TRIM31 from MAVS to weaken innate immunity and facilitate virus replication.

## DISCUSSION

**Fig 9:**
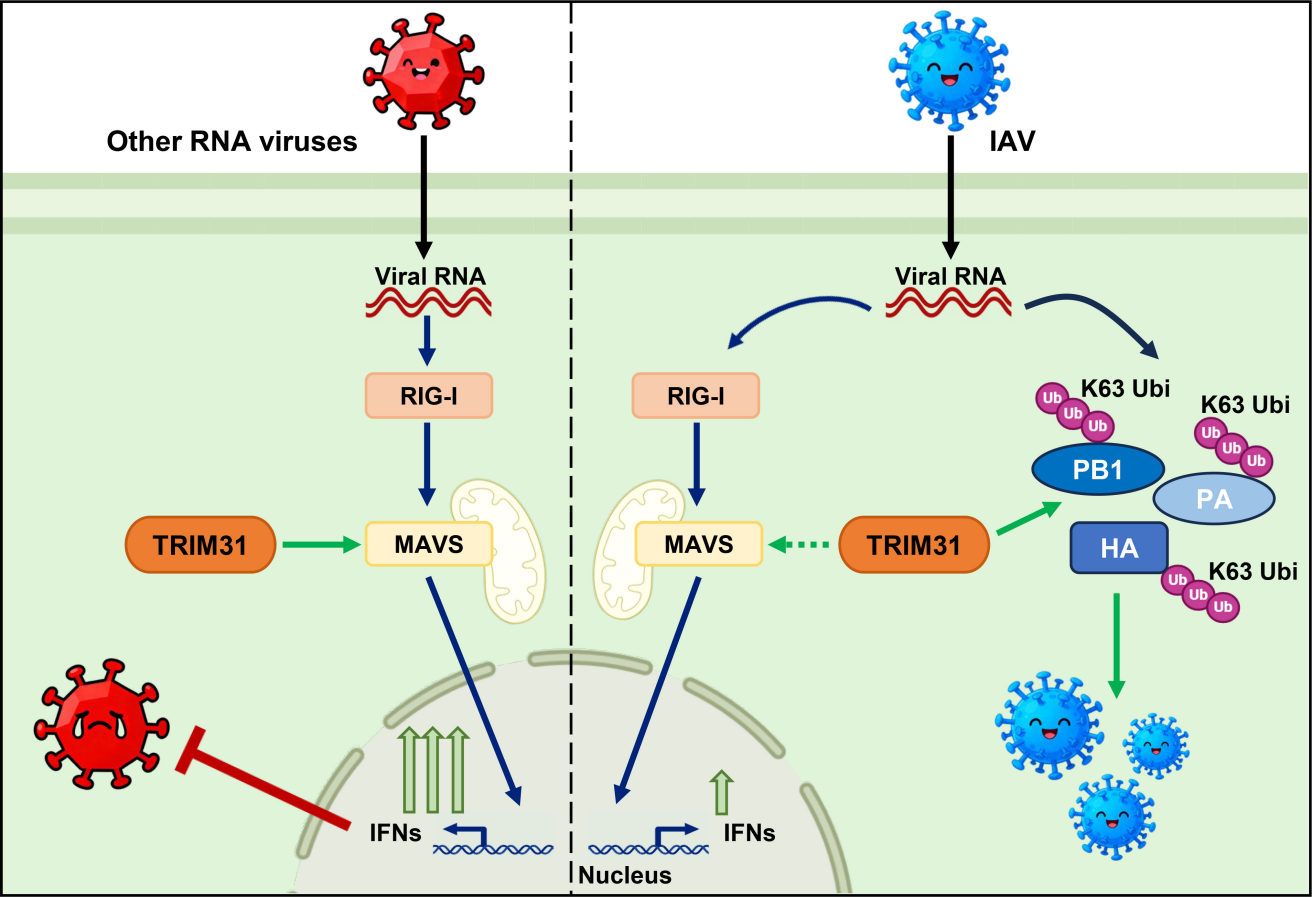
Working model of the distinct roles of TRIM31 in the replication of IAV and other RNA viruses. In cells infected with RNA viruses, such as SeV and VSV, TRIM31 promotes the MAVS-mediated IFN-I response and inhibits viral replication. Conversely, in IAV-infected cells, the PB1, PA, and HA proteins of IAV exploit TRIM31 to promote their stability and competitively bind to TRIM31 with MAVS, and thus counteract TRIM31’s antiviral effect on the IFN-I response. Blue arrows indicate MAVS-mediated type I IFN signaling pathway; green arrows indicate the process of TRIM31-mediated stabilization and activation of MAVS and viral proteins; and the dashed lines indicate the low-level interaction.

A previous study revealed that TRIM31 catalyzes the K63-linked ubiquitination of MAVS and promotes the MAVS-mediated IFN-I response, thereby inhibiting the replication of model viruses, such as SeV and VSV (26). In the present study, we identified a new role of TRIM31 in the life cycle of IAV (Fig. 9). Upon IAV infection, the PB1, PA, and HA proteins of IAV exploit TRIM31 to increase their stability by catalyzing K63-linked ubiquitination. The stabilized PB1, PA, and HA proteins competitively bind to TRIM31 with MAVS to attenuate the TRIM31-elevated IFN-I signaling. Therefore, IAV proteins exploit TRIM31 to fine-tune the inhibitory effect of TRIM31 on IAV replication. Finally, TRIM31, which is a previously known agonist of the IFN-I response, does not inhibit IAV replication as it does with SeV and VSV infections.

E3s constitute the central regulatory component of the ubiquitin-proteasome system and mediate the ubiquitination process through specific substrate recognition, thereby precisely controlling protein homeostasis by regulating stability, subcellular localization, and functional activity. Emerging evidence has demonstrated that certain E3s specifically target viral proteins, thereby modulating viral replication and pathogenicity through ubiquitination-dependent mechanisms. E3 TRIM21 has been shown to impede DNA replication of the hepatitis B virus by promoting the K48-linked ubiquitination and subsequent degradation of the viral DNA polymerase (32). S-phase kinase-associated protein 2 (Skp2) was recently identified as the probable E3 ubiquitin ligase responsible for the degradation of p24 and p55 of human immunodeficiency virus 1 (33). The E3 ligase RNF5 has been shown to restrict severe acute respiratory syndrome coronavirus 2 replication by catalyzing the ubiquitination and mediating the degradation of the E protein (34). In summary, these E3 ligases mostly act as restrictive factors against viral infections by catalyzing the ubiquitination and degradation of viral proteins. However, in the present study, we revealed that TRIM31 specifically catalyzes the K63-linked ubiquitination of the PB1, PA, and HA proteins of IAV, thereby strengthening the stability of the viral proteins.

TRIM31, as an E3 ligase, has been reported to be involved in multiple cell processes by catalyzing various types of ubiquitin chains on different substrates. For example, TRIM31 has been shown to regulate the K48-linked ubiquitination and degradation of p53, thereby promoting cancer cell resistance to anoikis and facilitating hepatocellular carcinoma progression (35). Conversely, in breast cancer, TRIM31 has been observed to induce the K63-linked ubiquitination of p53 and thus inhibit the proliferation, colony formation, migration, and invasion of breast cancer cells (27). TRIM31 has been shown to facilitate the K27-linked polyubiquitination of the non-receptor tyrosine kinase SYK at K375/571, promote the binding of SYK with C-type lectin receptors, and enhance antifungal immunity (31). Liu et al. demonstrated that TRIM31 serves as a positive regulator of MAVS-mediated innate antiviral immunity through the direct conjugation of K63-linked ubiquitin chains to the K10/311/461 sites on MAVS (26). The present study revealed the dual roles of TRIM31 in antiviral immunity and IAV infection. Although most of the aforementioned studies were conducted under varying pathophysiological conditions, these findings collectively indicate that TRIM31 plays vital but distinct roles in inhibiting or promoting disease progression in different diseases. However, we failed to identify the key sites on the viral proteins that mediate the TRIM31-catalyzed ubiquitination. Further studies are necessary to determine the specific sites or motifs by which TRIM31 catalyzes ubiquitination on the various viral proteins.

Viruses need to utilize host systems to assist in their replication, and IAVs are no exception (36). During their long commensalism with hosts, IAVs have evolved a complex set of mechanisms to regulate or hijack host systems for viral replication. However, due to the small segmented genome and limited encoding ability of IAV, IAV proteins, in addition to their unique functions in the virus life cycle, need to work in concert with each other to ensure smooth replication in host cells. In some cases, the formation of complexes by distinct viral proteins is imperative for the execution of specific functions. For example, three polymerase subunits of IAVs collaborate with NP to form the viral ribonucleoprotein complex (vRNP) to accomplish the replication and transcription of the viral genome (36). For the nuclear export of progeny vRNPs, the viral M1 protein binds directly to the vRNPs via the NEP protein to form the viral nuclear export complex (37). In other cases, multiple IAV proteins need to function separately to achieve the same effect. To effectively replicate in host cells, different IAV proteins target various host proteins in the innate immune signaling pathway to antagonize the host antiviral response. The non-structural protein 1 of IAV has been shown to suppress IFN production by targeting multiple host factors, including TRIM25, interferon regulatory factor 3 (IRF3), and 5′–3′ exoribonuclease 1 (38–40). Our previous study revealed that both the NP and PB1 protein of IAV mediate the degradation of MAVS and block the MAVS-mediated innate signaling pathway (5, 41). In the current study, we uncovered a novel pattern in which three different IAV proteins bind to the same target, TRIM31, in a synergistic manner, leading to the reduction of the MAVS-mediated IFN-I response elevated by TRIM31.

In summary, this study highlights the dual roles of TRIM31 in the life cycle of IAV. Upon IAV infection, TRIM31 catalyzes the K63-linked ubiquitination of MAVS and promotes the IFN-I response against virus infection. To counteract the antiviral effect of TRIM31, IAV exploits TRIM31 to catalyze the K63-linked ubiquitination of PB1, PA, and HA proteins, thereby enhancing the stability and functions of viral proteins. In addition, the stabilized viral proteins competitively interact with TRIM31 to inhibit the TRIM31–MAVS association and the MAVS-mediated IFN-I response. Taken together, IAV exploits TRIM31 to fine-tune its antiviral effect on the IFN-I response, thereby preventing TRIM31 from inhibiting IAV replication. Overall, our findings extend the knowledge of IAV–host interactions and reveal a key role of TRIM31 in the IAV life cycle.

## MATERIALS AND METHODS

### Cells, viruses, and plasmids

MDCK, HEK293T, BHK-21, PK-15, and U2OS were grown in DMEM (Gibco) supplemented with 10% (vol/vol) FBS (Gibco-BRL; 10099-141) and 1× penicillin/streptomycin (Gibco-BRL; 10378016). A549 cells were grown in Kaighn’s modified Ham F-12 nutrient mixture medium (Gibco) supplemented with 10% FBS and penicillin/streptomycin. All cells were cultured and maintained at 37°C with 5% CO_2_.

The H1N1 (A/Puerto Rico/8/1934, PR8), H1N1 (A/WSN/1933, WSN), and H6N6 (A/ duck/Hunan/4/2018, HN4) IAVs were stored in our laboratory. The H3N2 influenza virus (A/Swine/Shandong/TA05/2021, TA5) was kindly provided by Prof. Yihong Xiao (The Shandong Agricultural University, China). The H9N2 virus (A/chicken/Hunan/38/2018, HN38) was isolated from Gansu Province in China in 2018. Recombinant vesicular stomatitis virus expressing green fluorescent protein (VSV-GFP) was generated as described previously (42). The NDV (MG7 strain) was generated and stored in our laboratory (43). SeV (strain Fushimi) was kindly provided by Prof. Hongkui Deng (Peking University, China).

Human TRIM31, as well as TRIM31 truncations and C53/56A mutation, was constructed into pRK vector and pET-28a prokaryotic expression vector by using standard molecular biology techniques. The sh-TRIM31 off-target nonsense mutants of wild-type TRIM31, TRIM31-ΔRING, and TRIM31-C53/56A were constructed as shown in Fig. S2A. The genes encoding PB2, PB1, PA, and NP of PR8 or other viruses were amplified and then cloned into the pCAGGS vector. Flag-tagged PB1, PA, and HA from PR8-H1N1, SC15-H5N6, and SZ19-H7N9 were constructed and stored in our laboratory. The plasmid pPol I-Luc, for the expression of a viral RNA-like firefly luciferase gene under the control of the human RNA polymerase I promoter, has been reported previously (44). The plasmids of the IFN-β-Luc and pRL-TK internal control luciferase reporter plasmids used in the study were described previously (6).

### Reagents and antibodies

The antibodies used in this study were as follows: HRP-conjugated anti-Flag (A8592) (sigma), anti-HA (12013819001), anti-Myc (11814150001), and anti-GFP (11814460001) antibody (Roche); rabbit anti-PB2 (GTX125926), anti-PB1 (GTX125923), anti-PA (GTX118991), anti-HA (GTX640908), anti-NA (GT288), anti-M1 (GTX125928), anti-M2 (GTX125951), anti-NS1 (GTX125990), and anti-NS2 (GTX125953) polyclonal antibodies (Genetex); anti-TRIM31 (12543-1-AP) and anti-MAVS (14341-1-AP) (Proteintech); anti-β-Actin (TA-09), anti-GST-tag (TA-03), and HRP-conjugated goat anti-rabbit IgG (ZB-2301) (Zsbio); TBK1 (3013S), IRF3 (4302S), phosphorylated TBK1 (5483), IRF3 (4961) antibodies (Cell Signaling Technology); HRP-conjugated goat anti-mouse secondary antibody (ab102448), anti-IFIT1 (ab305301), anti-IFNAR1 (ab124764), anti-ubiquitin (ab134953), anti-ubiquitin (K63) (ab179434) and anti-GAPDH (ab181602) antibodies (Abcam); and Alexa Fluor 488-conjugated anti-mouse IgG (A0428), Cy3-labeled goat anti-rabbit IgG (A0516, Beyotime); the anti-IAV-NP nanobody (C7), fused with human IgG Fc fragment, was selected and produced based on previous descriptions (45).

Anti-Flag agarose affinity beads (A2220) and protein A/G agarose affinity beads (P6486/E3403), TPCK (T1426) (Sigma); CHX (ab120093) (Abcam); human IFN-β DuoSet ELISA kit (DY814-05) (R&D); Glutathione Sepharose 4B (17-0756-05) (GE Healthcare); Nano-Glo (N1120) (Promega); Polybrene (H9268), Poly(I:C) (P9582) and puromycin dihydrochloride (540222) (Merck); NP-40 (ST366), DAPI (C1002), recombinant human IFNα2a (P5646), normal rabbit IgG (A7016), and Cell Mitochondria Isolation Kit (C3601) (Beyotime). The scrambled negative control RNA (NC) and TRIM31-specific short interfering RNA were purchased from RiboBio Co. (China). Transfection reagent was purchased from Poly plus (China), TRIzol was obtained from Invitrogen (USA). SYBR Green I Master Mix was purchased from Roche (Germany).

### Viral infection

Viral infection was performed as previously described (44). All cells were seeded at the desired density in culture plates as per the requirements for different experiments. Viruses were inoculated into cells at a specific multiplicity of infection (MOI) for different experiments. One hour after inoculation, the medium was replaced with fresh OPTI-MEM, and the cells were incubated at 37°C. Virus-containing culture supernatants were collected at the indicated time points for titration.

### Virus titration

Virus titration was performed as previously described (45). Virus titers of virus stocks and cell culture supernatant were determined by end-point titration in MDCK cells. For the IAV, the supernatants were diluted and then used to infect confluent MDCK cells cultured in 12-well plates. At 1 h post-infection, supernatant was removed, and 1% methylcellulose with 1% TPCK-treated Trypsin was overlaid. At day 3 post-infection, overlay was removed, and cells were fixed with 4% formaldehyde for 1 h and stained with 1% crystal violet in 20% methanol. Plaques were counted by Image J, averaged, and multiplied by the dilution factor to determine viral titer as PFU/mL (46).

For the NDV and VSV-GFP, virus titers of virus stocks and cell culture supernatant were determined by end-point titration in MDCK cells. For end-point viral titration in MDCK cells, 10-fold serial dilutions of each sample were inoculated into MDCK cells. Infectious virus titers are reported as log_10_ TCID_50_/0.1 mL and were calculated from three replicates by the method of Reed–Muench (47).

### RNA interference

The RNA interference was performed as previously described (48). SiRNA targeting TRIM31 and scrambled siRNA (si-NC) were purchased from RiboBio Co. (China); the sequences of the siRNAs are listed in Table S1. All transfections with siRNA were performed as per manufacturer’s instructions using Lipofectamine RNAiMAX reagent.

Short hairpin RNA (shRNA) constructs were designed and cloned into the pLKO.1-EGFP-puro lentiviral backbone as per the manufacturer’s protocol (Tsingke Biotechnology, China). The target sequences for TRIM31 were listed in Table S1. The sequence of nonsense shRNA was provided by Tsingke. Lentivirus was produced in HEK293T cells transfected with viral constructs along with psPAX2 and pMD2G constructs. Viral supernatants were collected on days 2 and 3 after transfection and used to infect target cells.

### RNA isolation and qPCR

Total RNA from cells was extracted with TRIzol as previously described (48). For mRNAs, total RNA was subsequently transcribed into cDNA using M-MLV Reverse Transcriptase, according to the manufacturer’s protocol (Promega). GAPDH and β-actin were used as control for the normalization of cellular mRNA. Real-time PCR was carried out using the LightCycler 480 II (Roche). The RNA level of each gene was shown as the fold induction (2^-ΔΔCT^) in the graph. The sequences of the primers used for qPCR are shown in Table S1.

### Dual-luciferase reporter assays

For the viral minigenome assay, HEK293T cells were transfected with pCAGGS constructs expressing viral PB2, PB1, PA, and NP from PR8 virus, the construct pPol I-Luc, and an internal control pRL-TK (Promega), along with other plasmids or siRNA. Cells were incubated at 37°C for 24 h, and cell lysates were subsequently prepared by using the Dual-Luciferase Reporter Assay System (Promega). To detect activation of the IFN-I pathway, HEK293T cells were transfected with luciferase reporter plasmids (IFN-β-Luc) and the pRL-TK plasmid. At 24 h after transfection, the cells were left uninfected or infected with different viruses for 12 h, and cell lysates were subsequently prepared by using the Dual-Luciferase Reporter Assay System (Promega). The luciferase activities were measured on a GloMax 96 microplate luminometer (Promega), as reported previously (6).

### Western blotting and co-IP assay

The Western blotting and co-IP assay were conducted, as previously described (44). For Western blotting, cells were lysed in RIPA buffer (Beyotime, China). Proteins were separated by 10% SDS-PAGE and transferred to a nitrocellulose membrane (Bio-Rad). The membrane was blocked for 1 h in TBST containing 5% milk and subsequently incubated with primary antibodies for 2 h. After 1-h incubation with HRP-conjugated secondary antibody. The immunoreactive bands were visualized using an e-BLOT system (e-BLOT Life Science, China). The intensities of the target bands were quantified by using the Image J program (NIH, USA).

HEK293T cells were co-transfected with the indicated plasmids with or without virus infection for 24 h. The transfected cells were then harvested and lysed in NP-40 lysis buffer (20 mM Tris-HCl [pH 7.5], 150 mM NaCl, 1% NP-40, 1 mM EDTA with protease inhibitor cocktails). For each immunoprecipitation, 1 mL of lysate was incubated for 4 h at 4°C with 0.5 µg of the indicated antibody or control IgG and 30 µL of protein A/G-Sepharose (Sigma). The beads were washed three times with 1 mL of lysis buffer containing 500 mM NaCl. The precipitates were analyzed by using standard immunoblotting procedures.

### GST pull-down assay

GST pull-down assays were conducted as previously described with slight modifications (49). Briefly, the GST-tagged fusion proteins and the control GST proteins were expressed in BL21 cells after induction with 0.1 mmol/L IPTG overnight at 18°C. Centrifuged cells were resuspended in lysis buffer (1× PBS, 0.2 mM PMSF, 1% Triton X-100) and sonicated for 15 min. After centrifugation, the supernatant was applied to a Glutathione–Sepharose 4B bead column (GE Healthcare), in accordance with the manufacturers’ instructions. Purified GST-tagged fusion proteins were diluted with 1× PBS and filtered through Amicon Ultra 0.5 mL filters (Millipore). Then, 1 µg of purified GST protein or GST fusion protein was captured by the Glutathione–Sepharose 4B beads (GE Healthcare). The beads were then washed three times with ice-cold PBS. The supernatant was loaded onto gels, followed by immunoblotting analysis.

### Confocal microscopy

Confocal microscopy was performed as previously described (49). Cells were seeded in 12-well plates (5 × 10^5^ cells/well) on coverslips. After transfection or infection at indicated times, cells were then fixed with 4% paraformaldehyde for 20 min at room temperature and washed three times with PBS. Cells were permeabilized with 0.1% Triton X-100 in PBS for 10 min and blocked with 5% skimmed milk for 1 h. Cells were incubated with the indicated primary and secondary antibodies or not. Then, the cells were stained by DAPI before observation by ZEISS microscope (LSM 980) with a 100× oil objective. The colocalization analysis was conducted with ImageJ software. The data were further analyzed by using GraphPad Prism 8.

### Detection of ubiquitin-modified proteins

The experiments were performed as previously described (50). Briefly, the cells were lysed in lysis buffer containing 1% SDS and denatured by heating at 95°C for 10 min. After centrifugation, the supernatants were diluted with NP-40 lysis buffer until the concentration of SDS was 0.1%, and were then co-immunoprecipitated with the indicated antibodies. Ubiquitin-modified proteins were detected by immunoblotting with the indicated antibodies.

### Membrane fusion assay

The experiments were performed, as previously described (41). A549 cells were transfected with Flag-TRIM31 or EV for 24 h and then infected with PR8/H1N1 for 12 h. The cells were treated with trypsin in 2 µg/ mL for 15 min at 37°C to cleave HA into HA1 and HA2. After being washed, the cells were treated with citric acid (pH 5.0) for 15 min at 37°C. The acidic medium was replaced with DMEM supplemented with 10% FBS, and the cells were cultured for 3 h, followed by 4% polyoxymethylene fixation. Syncytium formation was observed by immunofluorescence with indicated antibodies and analyzed by counting the nuclei in syncytia in five random microscopic fields as previous study (51).

### Statistical analysis

Data are expressed as the mean ± standard deviation. Statistical significance was determined by using the Student’s two-tailed unpaired *t*-test or analysis of variance with GraphPad Prism software (version 8.0, San Diego, CA, USA). Differences between groups were considered significant when the *P*-value was < 0.05 (*), <0.01 (**), <0.001 (***), and <0.0001 (****); ns indicates no significant difference.

## Data Availability

All data are included in the article and supplemental material.

## SUPPLEMENTAL MATERIAL

Supplemental material jvi.01893-25-s0001.pdf Figures S1 and S2; Table S1.

ASM does not own the copyrights to Supplemental Material that may be linked to, or accessed through, an article. The authors have granted ASM a non-exclusive, world-wide license to publish the Supplemental Material files. Please contact the corresponding author directly for reuse.
