# Supplementary material for: The viral proteins of influenza A virus competitively bind to TRIM31 with MAVS to fine-tune the antiviral innate immunity
Source: J Virol. 2025 Nov 26;99(12):e01893-25. doi: 10.1128/jvi.01893-25 (PMC12724384; doi:10.1128/jvi.01893-25)
Supplement: Supplemental material — Figures S1 and S2; Table S1. [file jvi.01893-25-s0001.pdf]

Supplementary data for

The viral proteins of influenza A virus competitively bind to TRIM31 with MAVS to fine-tune the antiviral innate immunity

Running title: IAV proteins exploit TRIM31 to fine-tune IFN response

Jiaxin Huang[a,b], Shuai Xu[b], Junwen Liu[b], Qian Wang[b], Lu Han[b], Mengyao Ji[b], Caoqi Lei[d], Qiyun Zhu[a,b,*], and Hualan Chen[a,c,*]

[a] College of Veterinary Medicine, Gansu Agricultural University, Lanzhou 730070, China

[b] State Key Laboratory for Animal Disease Control and Prevention, Lanzhou Veterinary Research Institute, Chinese Academy of Agricultural Sciences, Lanzhou, 730000, PR China

[c] State Key Laboratory for Animal Disease Control and Prevention, Harbin Veterinary Research Institute, Chinese Academy of Agricultural Sciences, Harbin, 150069, PR China

[d] School of Basic Medical Sciences, Lanzhou University, Lanzhou 730000, PR China

* To whom correspondence should be addressed. Email: chenhualan@caas.cn or zhuqiyun@caas.cn

This file includes:

Figures S1 to S2

Table S1

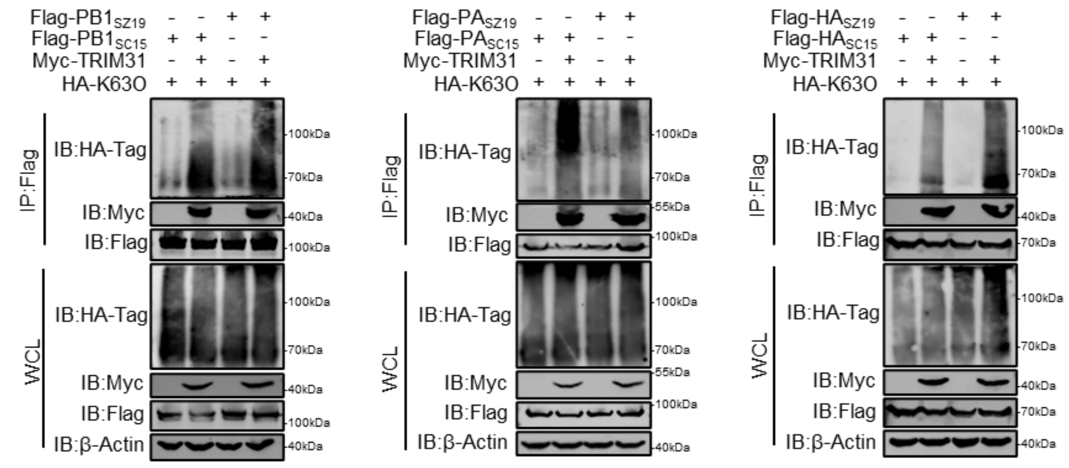

Fig. S1. TRIM31 promotes the K63-linked ubiquitination of PB1, PA, and HA proteins from H5N6 and H7N9 IAV. HEK293T cells were transfected with Myc-TRIM31, Flag-PB1$_{SC15}$, Flag-PB1$_{SZ19}$, Flag-PA$_{SC15}$, Flag-PA$_{SZ19}$, Flag-HA$_{SC15}$, Flag-HA$_{SZ19}$, and HA-K63O for 24 h, and then subjected to co-immunoprecipitation and immunoblotting analysis with indicated antibodies.

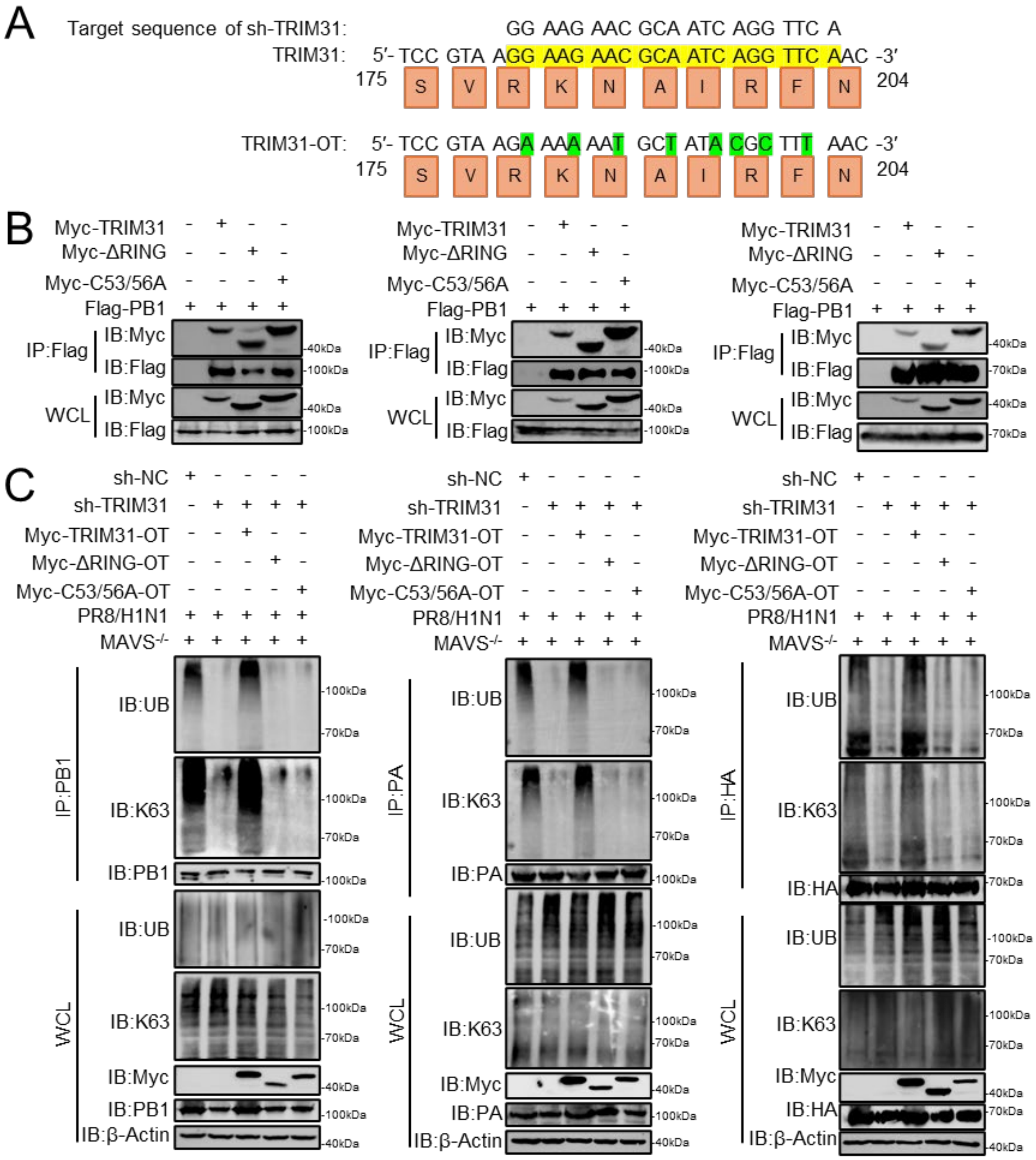

Fig. S2. The ubiquitin ligase activity of TRIM31 is critical for the stabilization of PB1, PA, and HA proteins. (A) The generation of sh-TRIM31 off-target nonsense mutants. The target sequences of sh-TRIM31 within the TRIM31 open read frame were replaced with serial nonsense mutations. The target sequence of shTRIM31 is highlighted in yellow. The mutated nucleotides are highlighted in green. (B) HEK293T cells were transfected with the indicated plasmids for 24 h before coimmunoprecipitation and immunoblot analysis. (C) Sh-TRIM31 or sh-NC MAVS-knockout HEK293T cells were transfected with TRIM31-OT, TRIM31-ΔRING-OT, TRIM31-C53/56A-OT, or EV for 24 h and then infected with WSN/H1N1 for 24 h. The cells were then used for ubiquitination assays.

Table S1. The siRNA, shRNA, and primers used in this study.

Name	Sequence （5' - 3'）
Control siRNA (si-NC)	N/A
si-TRIM31	AGAACGCAATCAGGTTCAA
sh-NC	CCGGGGTTCTCCGAACGTGTCACGTCTCGAGACGT GACACGTTCGGAGAACCTTTTTG
sh-TRIM31	CCGGGGAAGAACGCAATCAGGTTCACTCGAGTGAA CCTGATTGCGTTCTTCCTTTTTT
β-Actin-F	GTCGTCGACAACGGCTCCGGCATG
β-Actin-R	ATTGTAGAAGGTGTGGTGCCAGAT
GAPDH-F	GAGTCAACGGATTTGGTCGT
GAPDH-R	GACAAGCTTCCCGTTCTCAG
TRIM31-F	AACCTGTCACCATCGACTGTG
TRIM31-R	TGATTGCGTTCTTCCTTACGG
IFNB1-F	CATTACCTGAAGGCCAAGGA
IFNB1-R	CAATTGTCCAGTCCCAGAGG
ISG15-F	AGGACAGGGTCCCCCTTGCC
ISG15-R	CCTCCAGCCCGCTCACTTGC
OASL-F	CTGATGCAGGAACTGTATAGCAC
OASL-R	CACAGCGTCTAGCACCTCTT
CXCL10-F	GTGGCATTCAAGGAGTACCTC
CXCL10-R	TGATGGCCTTCGATTCTGGATT
RANTES-F	CCTGCTGCTTTGCCTACATTGC
RANTES-R	ACACACTTGGCGGTTCTTTCGG

N/A: not available.
